# A Charge-Transfer-Induced Strategy for Enantioselective Discrimination by Potential-Regulated Surface-Enhanced Raman Scattering Spectroscopy

**DOI:** 10.3390/bios13040471

**Published:** 2023-04-12

**Authors:** Yue Wang, Yucong Liu, Chunyu Ren, Ruofei Ma, Zhangrun Xu, Bing Zhao

**Affiliations:** 1 Department of Chemistry, College of Sciences, Northeastern University, Shenyang 110819, China; 2 State Key Laboratory of Supramolecular Structure and Materials, Jilin University, Changchun 130012, China

**Keywords:** enantioselective discrimination, charge transfer, electrochemical SERS, potential regulation

## Abstract

A simple and efficient enantioselective discrimination method, especially the chirality-label-free discrimination method, for the recognition of chiral small molecules with high resolution and wide applicability has been urgently desired. Herein, achiral Au/*p*-aminothiophenol (PATP) substrates were prepared to link the enantiomers via coupling reactions for constructing the enantioselective discrimination system. The resultant Au/PATP/enantiomer systems displayed charge-transfer (CT)-induced surface-enhanced Raman scattering (SERS) spectra that offered distinguishable information for the systems with different chirality. The differentiated spectral signal can be amplified by regulating the applied electrode potential, leading to great enantioselective discrimination performance. Moreover, the relationship between the discrimination performance and the potential-regulated CT process was revealed by SERS, which enabled an accurate and effective enantiomeric determination for various chiral molecules, including aromatic and aliphatic small molecules. The aliphatic molecule with the shorter chain was discriminated with a higher resolution, since the longer-chain molecule in the discrimination system may cause a change in the molecular electronic structure of the PATP. In addition, the aromatic chiral molecule can be distinguished easier than the aliphatic molecules, which means that the generation of the conjugation of electrons in the aromatic molecule-involved enantiomeric systems facilitates CT-induced SERS discrimination. Our work provides guidance for the design and development of an effective enantioselective discrimination strategy with high discrimination performance in diverse application fields.

## 1. Introduction

Chirality exists widely in nature, and chiral compounds are closely associated with human daily life [1,2]. Many biomolecules, pharmaceuticals, personal care products, and agrochemicals are chiral [3]. Considering that stereoselectivity is involved in many biological processes of organisms and that the two enantiomers of one chiral molecule generally possess distinctly different biological activities, enantioselective discrimination is of great significance in various fields. For example, amino acids, the essential building blocks of the human body, are regarded as an indicator of many diseases and frequently used as components of food and pharmaceutical products [4,5]. The discrimination of amino acid enantiomers, such as phenylalanine (Phe), phenyllactic acid (PLA), phenylglycine (Phg), and other aromatic amino acids, is of great importance since many amino acid enantiomers are used as crucial intermediates for the synthesis of antibiotics, antiviral, or antitumour pharmaceuticals [6]. Furthermore, they work as additives to improve the nutrition and flavor in food products [7]. As another example, the determination of the level of the enantiomeric form of branched-chain amino acids, including valine (Val) and leucine (Leu), is important to evaluate the obesity and insulin resistance of diabetics [8]. In addition, the enantiomers of such branched-chain amino acids are widely used as nutritional additives in the food industry [9]. In this respect, a universal and sensitive enantioselective discrimination method for chiral molecules, especially various amino acids and amino acid derivatives, is especially important. However, chiral small molecules are generally difficult to discriminate with high resolution. Up to now, numerous methodologies have been applied to the discrimination of a pair of enantiomers, including chromatography, spectroscopy, the electrochemistry method, and the colorimetric method [10,11]. Among these methods, the feasibility of enantioselective discrimination based on stereospecific interaction is highly dependent on the affinity of chiral selectors, such as chiral columns, chiral electrodes, and chiral substrates [12,13]. The chiral selectors with the proper structure and molecular polarity are indispensable for sensitive and specific enantioselective discrimination. In the case of the methods that rely on circularly polarized light, such as circular dichroism, vibrational circular dichroism, and Raman optical activity spectroscopy methods, the capability of enantioselective discrimination is commonly affected by the optical response between the chiral light and the enantiomers [14,15]. Moreover, the low sensitivity of such chiral spectroscopy methods generally restricts their application to various chiral molecules. Therefore, an effective enantiomeric discrimination method with high resolution and sensitivity that is applicable to a variety of enantiomers is still in demand but a challenge.

Recently, we have developed chirality label-free surface-enhanced Raman scattering (SERS) strategies for enantioselective discrimination of chiral small molecules, in which neither the chiral selector nor chiral light is indispensable [16,17]. In these label-free methods, the difference in the intermolecular interaction of the enantiomers with the achiral Raman probes is the origin of the SERS enantioselective discrimination, which causes different charge transfer (CT) processes between the two enantiomeric systems. The differentiated SERS spectral signals of the two enantiomeric systems are produced, and then the chirality of the target molecule can be distinguished. These CT-induced chirality-label-free SERS strategies exhibit superiority in the combination of universality and sensitivity in comparison with the traditional chirality-labeled strategies, since CT enhancement is one of the main and generally accepted mechanisms for SERS [18]. The recognition efficiency is highly dependent on the CT properties of the enantiomeric systems, which also greatly affect the SERS signal in the systems. These methods are not limited to the recognition of the specified enantiomers but are available for diverse enantiomers, including chiral alcohols, chiral amines, chiral acids, and amino acids, by constructing plasmonic nanosubstrates with different CT properties [16,17,19]. Tuning the CT process in the enantiomeric discrimination system is expected to enable the improvement of the performance of enantiomeric discrimination.

Regulating the energy level of the plasmonic metal is an effective means to change the CT transitions at the metal-molecule interface [20]. It is reasonable to tune the Fermi level of the metal in an electrochemical system by altering the electrochemical potential of the electrode that is modified with the plasmonic nanosubstrates. When the applied potential on the electrode is changed, the surface charge distribution of the electrode changes, which influences the energy of the Fermi level on the electrode [21]. In addition, the change in the applied potential of the electrode also influences the interaction between the metal electrode and the adsorbed molecules [22]. The CT transition generating between the Fermi level of the metal electrode and the molecular orbital level of the adsorbed molecules changes accordingly, which can exert a great impact on the SERS vibrational modes related to the Herzberg-Teller type CT contribution [23]. Therefore, tuning the electrochemical potential may be an effective way to improve the CT-induced enantioselective discrimination performance. 

Au nanoparticles (NPs) are one of the conventional and intriguing plasmonic nanomaterials with excellent surface plasmon resonance (SPR) effects, owing to the fact that their optical transitions are located in the visible range of the spectrum with strong intensity [24,25]. Au NPs can effectively promote the generation of energetic hot electrons and significantly enhance the local electromagnetic field when they are excited by visible light [26]. Such a local electromagnetic field produced by Au NPs contributes to the strong SERS intensities, which render Au NPs as remarkable SERS active substrates for detection and sensing [27,28]. In the field of electrochemical analysis, Au-based nanomaterials also exhibit superiority due to their high electrical conductivity, easy functionalization, and good chemical stability even under a wide range of pH, temperature, and ionic strength [29]. Moreover, the energy of the Fermi level of the Au-based electrodes can be easily regulated by controlling the applied electrode potential, leading to the tunable energy level alignment and the CT process at the electrode-adsorbates interface [30]. However, the relationship between the applied potential of the plasmonic Au nanosubstrates and the chirality-label-free SERS enantioselective discrimination remains to be investigated for high chiral discrimination performance. 

In this work, Au NPs modified indium tin oxide (ITO) electrodes with the surface decoration of a monolayer of *p*-mercaptopyridine (MPY) molecules were prepared and used as chirality-label-free substrates to investigate the effect of electrochemical potential on the discrimination performance of the CT-based SERS enantiomeric discrimination method. The enantioselective discrimination signals of the two enantiomeric systems were obtained by electrochemical SERS measurements under the application of different electrochemical potentials. The discrepancy-amplified chirality-label-free SERS enantioselective discrimination strategy was thus proposed, in which various chiral small molecules, including aromatic and aliphatic small molecules, can be distinguished with high resolution. Our work provides a basic understanding of the relationship between the discrimination performances of the enantiomers and the electrochemical potential-induced CT process and is instructive for the design and development of universal enantioselective discrimination strategies with high performance. 

## 2. Materials and Methods

### 2.1. Reagents

The enantiomers of chiral molecules, including Phe, Phg, mandelic acid (MA), and the Raman probe molecule *p*-aminothiophenol (PATP), were purchased from J&K Scientific Ltd. (Beijing, China). The enantiomers of chiral molecules such as PLA, 2-hydroxy-4-phenylbutyric acid (HPBA), Val, Leu, and 2-amino-5-methylhexanoic acid (AMHA) were obtained from Macklin Biochemical Co., Ltd. (Shanghai, China). Poly (diallyldimethylammonium chloride) solution (PDDA, 50 wt.%), 1-ethyl-3-(3-dimethyl aminopropyl) carbodiimide (EDC), and N-hydroxysuccinimide (NHS) were acquired from Aladdin Reagent Co., Ltd (Shanghai, China). Chloroauric acid tetrahydrate (HAuCl_4_·4H_2_O), trisodium citrate dihydrate, potassium dihydrogen phosphate (KH_2_PO_4_), potassium phosphate dibasic (K_2_HPO_4_), potassium chloride (KCl), and all the other chemical reagents were purchased from Sinopharm Chemical Reagent Co., Ltd. (Shanghai, China). Distilled and deionized water from a Milli-Qplus system with a resistivity greater than 18 MΩ·cm was used throughout the experiment.

### 2.2. Instruments and Characterization

UV-vis spectra were measured using a UV-1800PC spectrophotometer by recording the absorption spectra. The hydrodynamic diameter of the NPs was collected by employing a Malvern particle sizer (Nano ZS90, Malvern, UK). The morphology and size of the Au NPs were taken by a field emission transmission electron microscope (TEM, TECNAI G20, FEI, Hillsboro, OR, USA). The scanning electron microscope (SEM) images were characterized using a Hitachi SU8010 field-emission system operated at 10.0 kV. Electrochemical measurements were performed on a CHI 600E electrochemical workstation (Shanghai Chenhua, China).

### 2.3. Preparation of PATP Functionalized Au NPs Substrates

The Au NPs were synthesized by the reduction of HAuCl_4_ with trisodium citrate, as previously described in the literature [31]. The Au NPs substrates were prepared by the self-assembly of Au NPs on the surface of a hydroxylated ITO-coated glass based on electrostatic interaction. The hydrophilic treatment of ITO glass substrates was performed by using an aqueous solution containing ammonium hydroxide and hydrogen peroxide [32]. Furthermore, the hydroxylated ITO glass was immersed in PDDA solution for 30 min for surface charge modification. After being rinsed and dried, the positively charged PDDA was adhered to the surface of the ITO glass, which could adsorb the negatively charged Au NPs by being soaked in the colloidal Au NPs. A layer of Ag NPs was assembled on the surface of ITO glass slide and the Au NPs substrates were complete. The prepared Au NPs substrates were immersed in an ethanol solution of 10^−2^ M PATP for 2 h, and then rinsed by ethanol and dried by nitrogen. After the surface functionalization of the Au NPs substrates with PATP, the achiral Au/PATP substrates were achieved.

### 2.4. Constructing of Chirality-Label-Free Enantiomeric Discrimination Systems

**Figure 1:**
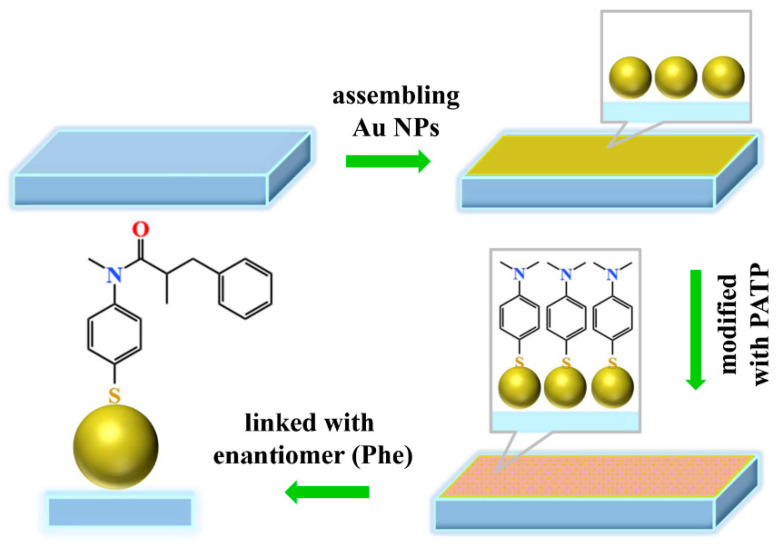
Schematic of the preparation process of the chirality-label-free enantioselective discrimination system, taking chiral molecule Phe as an example.

First, 10^−2^ M solutions of different enantiomeric molecules were prepared by using a 0.1 M phosphate buffered saline (PBS) buffer solution. A 0.20 mM EDC solution and a 0.50 mM NHS solution were added to the above enantiomeric solution with a volume ratio of 1:1:9. The mixture was stirred at 37 °C for 20 min to activate the carboxylic acid groups of the enantiomeric molecules. Furthermore, the prepared achiral Au/PATP substrates were incubated in the mixed solutions containing the different activated enantiomeric molecules at 37 °C for 2 h. After rinsing with PBS buffer solution to remove the unreacted reagents, the Au/PATP/enantiomer discrimination systems were finally constructed. The preparation process and the structure of the achiral Au/PATP substrates and the chirality-label-free enantioselective discrimination system (taking the Au/PATP/Phe system as an example) were schematically illustrated in Figure 1.

### 2.5. SERS Measurement

SERS measurements were performed using a confocal Raman microscope (XploRA ONE, Horiba Jobin Yvon, Paris, France) with a laser wavelength of 638 nm. The laser was focused on the surface of the sample through a 50× long-distance objective lens with a 1 μm spot size. All SERS spectra were obtained under the same conditions with an acquisition time of 5 seconds and a power of 7.4 mW using a holographic grating of 1200 grooves/mm. The Raman band of a silicon wafer at 520.7 cm^−1^ was used to calibrate the spectrometer. The SERS mapping images were obtained with 50× long-distance objective lens, a scanning step of 0.3 μm, and an exposure time of 1 s at 638 nm excitation.

### 2.6. Electrochemical SERS Measurement

Electrochemical SERS measurement was carried out by a three-electrode system in an electrolyte of 0.1 M PBS solution (pH 7.0) at room temperature. The ITO glasses modified by the Au/PATP/enantiomer discrimination systems with a bare surface area of 1.0 cm^2^ were used as the working electrode (WE). A Pt wire (0.5 mm diameter) and a silver/silver chloride (Ag/AgCl) wire (4 mm diameter) were used as the counter electrode (CE) and the quasi-reference electrode (RE). The electrochemical SERS measurements were performed at different potentials applied from −0.5 to 0.4 V (vs Ag/AgCl). Cyclic voltammetry (CV) measurements were taken at a scan rate of 50 mV/s in the range of −0.5 to 0.5 V potential.

## 3. Results and Discussion

### 3.1. Characterization of the Achiral Au/PATP Substrates and Au/PATP/Enantiomer Discrimination Systems

**Figure 2:**
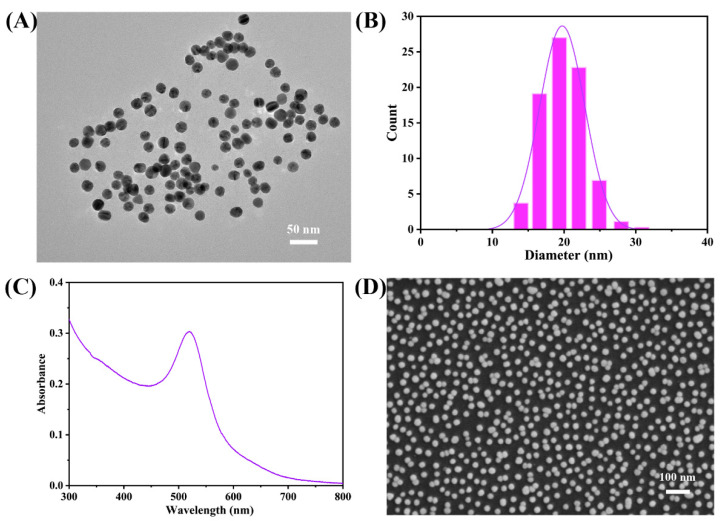
TEM image (**A**), the size distribution (**B**), and UV-vis absorption spectrum (**C**) of the prepared Au NPs. (**D**) SEM image of the surface morphology of the assembled Au NPs substrate.

**Figure 3:**
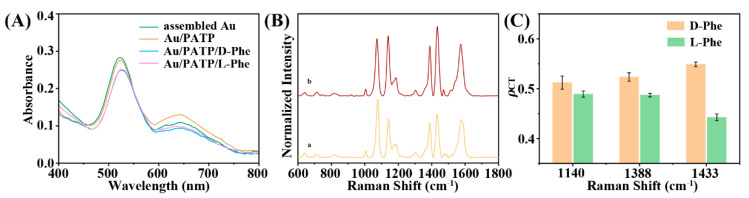
(**A**) UV−vis absorption spectra of the assembled Au NPs, Au/PATP, Au/PATP/D−Phe, and Au/PATP/L−Phe. (**B**) SERS spectra of the discrimination systems Au/PATP/D−Phe (curve b) and Au/PATP/L−Phe (curve a). (**C**) *ρ*_CT_ of 1140, 1388, and 1433 cm^−1^ bands of PATP in the D and L−Phe enantiomeric systems.

The prepared colloidal Au NPs are spherical and monodisperse with a uniform particle size, and their morphology is displayed in the TEM image in Figure 2A. The average diameter of the Au NPs s about 20 nm, which is consistent with the hydrodynamic diameter measured by dynamic light scattering (DLS) (Figure 2B). The UV-vis spectra showed the sharp SPR absorption of prepared Au NPs at around 520 nm (Figure 2C). The Au NPs substrates were fabricated by assembling Au NPs on the surface of the ITO glass based on the electrostatic interaction between the positive PDDA and the hydroxylated ITO glass. The SEM image in Figure 2D indicates that the Au NPs are homogeneously assembled on the ITO glass to form an Au NPs monolayer film, in which no obvious aggregation between the Au NPs is observed. The SPR absorption of the Au NPs immobilized on the ITO glass substrates shifted from 520 nm to 524 nm due to the dipole-dipole electromagnetic interaction, as shown in Figure 3A [33]. When a layer of PATP molecules was decorated on the Au NPs substrates, no obvious change occurred in the SPR adsorption of the PATP-modified Au NPs substrates. After the Phe enantiomers were crosslinked on the Au/PATP substrates to construct the enantiomeric discrimination systems, a distinct redshift of the SPR absorption of the Au NPs to 532 nm could be observed. Such redshift can be a demonstration for the successful immobilization on the Au/PATP substrates with the enantiomeric molecules, which is because of the modification of the dielectric environment of the Au NPs [34]. However, the enantiomers with different chirality produced a negligible difference in the SPR absorption of the Au NPs between the two enantiomeric discrimination systems, indicating the poor applicability of UV-vis spectroscopy for the chirality-label-free enantiomeric discrimination.

### 3.2. SERS Reproducibility and Discrimination Performance of the Achiral Au/PATP Substrates

Good reproducibility of the Au/PATP substrates is vital for reliable and high-performance chirality-label-free SERS enantioselective discrimination. To verify the reproducibility of such Au/PATP substrates, we collected a total of 625 SERS spectra across a selected area of 10 × 10 µm^2^, and the mapping images were integrated by the SERS bands of PATP at 1705 and 1140 cm^−1^, assigned to totally symmetric (a_1_) mode and the non-totally symmetric (b_2_) mode, respectively (Figure S1, Supplementary Materials). There is no remarkable change in the SERS spectral intensity between different collection points. The calculated relative standard deviation (RSD) values of the band intensities at 1705 and 1140 cm^−1^ were 4.7% and 4.0%, respectively, demonstrating the great reproducibility of the Au/PATP substrates. Subsequently, D- and L-Phe were chosen as the target molecules to investigate the enantioselective discrimination performance of the achiral Au/PATP substrates by SERS. All the SERS spectra of the enantiomeric discrimination systems were normalized by the strongest band of PATP at 1075 cm^−1^, attributed to the C-S stretching vibration mode [35,36]. As expected, the achiral substrates exhibit great enantioselective discrimination performance. As shown in Figure 3B, the intensities of the b_2_ modes of PATP at 1140, 1388, and 1433 cm^−1^, assigned to C-H bending vibration, the coupling vibrations of C-H rocking and C-C stretching, and the coupling vibrations of C-C stretching and C-H rocking, in the Au/PATP/L-Phe system were lower than those in the Au/PATP/D-Phe system. The difference in the b_2_ modes of the two enantiomeric systems can be an indicator for the occurrence of different CT processes between the L-Phe and D-Phe systems [16,37]. Such difference in the intensities of the b_2_ modes is only dependent on the chirality of the enantiomeric system, which caused the differentiated SERS spectra. The suggested reason for the different CT processes between the two enantiomeric discrimination systems can be attributed to the different intermolecular interactions that originate from the molecular chirality between the two enantiomers and the probe molecule PATP [16,17]. The CT transition from the Fermi level of Au to the molecular orbital level of PATP was facilitated in the Au/PATP/D-Phe system compared with that in the Au/PATP/L-Phe system. The degree of CT (*ρ*_CT_) values, proposed by Lombardi et al. [38], were calculated to estimate the difference of CT resonance between the two enantiomeric systems. The *ρ*_CT_ of a k-spectral bond can be determined using Equation (1):

(1) $$\rho_{\mathrm{CT}\left(k\right)}=\frac{I_{\left(\mathrm{CT}\right)}^{k}-I_{\left(\mathrm{SPR}\right)}^{k}}{I_{\left(\mathrm{CT}\right)}^{k}+I_{\left(\mathrm{SPR}\right)}^{0}}$$

The k-spectral bond is used to identify the bond in a SERS spectrum, in which CT resonance makes an additional contribution to its SERS intensity except for the contribution of SPR. While the bond with the index “0” is the a_1_ mode with only contributions from SPR, with intensity denoted *I*^0^_(SPR)_. For a b_2_ mode in a SERS spectrum, *I^k^*_(SPR)_ is usually quite small or zero, which means that the main contribution to the SERS intensity arises from CT. In the enantioselective discrimination SERS spectra, the a_1_ mode at 1075 cm^−1^, whose intensity was regarded as *I*^0^_(SPR)_, and the b_2_ modes at 1140, 1388, and 1433 cm^−1^, whose intensities were regarded as *I^k^*_(CT)_, were chosen to calculate the *ρ*_CT_ values. As shown in Figure 3C, the calculated *ρ*_CT_ values of the two enantiomeric discrimination systems further demonstrated the existence of differentiated CT processes in the two enantiomeric systems. The above SERS results indicated that such achiral Au/PATP substrates can effectively distinguish the two enantiomers with different chirality.

### 3.3. Electrochemical SERS Enantioselective Discrimination for the Au/PATP/Enantiomer Discrimination System

Considering that the CT process occurring at the electrode surface can be tuned by changing the electrode potential, SERS spectra of the Au/PATP/L-Phe and Au/PATP/D-Phe enantiomeric systems under different applied potentials were measured. To investigate the stability of the Au/PATP/enantiomer electrode in the three-electrode system, CV measurements were performed, in which the electrode potential was swept between −0.5 and 0.5 V in the PBS solution. The CV curves of the Au substrate, Au/PATP, and the Au/PATP/D-Phe and Au/PATP/L-Phe enantiomers are shown in Figure S2, respectively. A negligible change is observed for the shape of the CV curve of the Au/PATP electrode compared with that of the Au NPs electrode, indicating that neither an electrochemical redox reaction nor the desorption of the PATP molecules occurred at the surface of the Au/PATP electrode under the applied potential conditions. When the D- and L-Phe enantiomers bonded with the achiral Au/PATP substrate separately, no oxidation peak or reduction peak appeared. Except for the decrease in the current intensity, there was no change in the shape of CV curve of the Au/PATP/D-Phe or Au/PATP/L-Phe electrodes in comparison with that of the Au/PATP electrode. The possible reason for the decrease in the current is the reduction of the conductivity after the modification of Phe enantiomers, which is also evidence for the successful construction of the Au/PATP/Phe enantiomer system.

**Figure 4:**
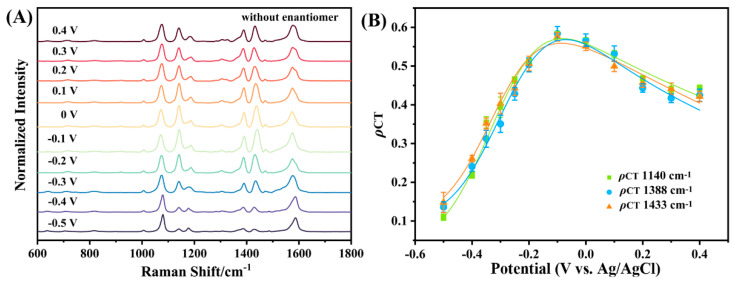
(**A**) Normalized potential-dependent SERS spectra of the Au/PATP substrate without any enantiomers. The applied potential is from −0.5 to 0.4 V. (**B**) *ρ*_CT_ profiles of 1140, 1388, and 1433 cm^−1^ bands of PATP in the Au/PATP system.

Electrochemical potential-dependent SERS spectra of the Au/PATP substrate with and without Phe enantiomers were recorded under different applied potentials from −0.5 to 0.4 V with a potential interval of 0.1 V, which does not cause either the redox reaction or the desorption of the adsorbed PATP on the electrode. As shown in Figure 4, the SERS intensity of each system changed with the applied potential from the negative to the positive potential. The intensities of the b_2_ modes of PATP in the Au/PATP system increased first as the potential reached −0.1 V potential, and then decreased with the regulation of the applied potential to 0.4 V (Figure 4A). While no considerable change in the intensity of the a_1_ mode was observed in this potential region (Figure S3), this indicated that the molecular orientation of the adsorbed PATP in the systems was not significantly affected by the applied potential [39]. The potential-dependent changes in the SERS intensity of the Au/PATP electrode may originate from the change in the Au/PATP interfacial interaction and the Fermi level of the Au NPs substrates. The corresponding *ρ*_CT_ values of the Au/PATP system under the influence of electrode potential were plotted in Figure 4B. An asymmetric, potential-dependent *ρ*_CT_ profile of the 1140, 1388, and 1433 cm^−1^ bands of PATP in the Au/PATP system was obtained. Such an asymmetric curve of *ρ*_CT_ versus potential as a result of Fano interference is regarded as the characteristic feature of CT enhancement [40,41].

**Figure 5:**
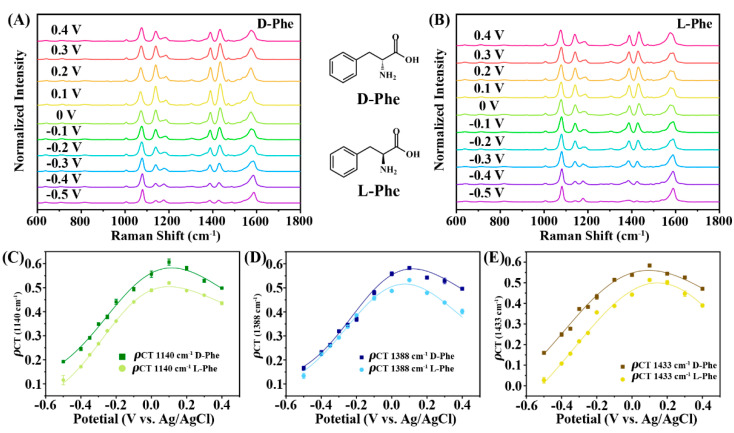
Normalized potential−dependent SERS spectra of the (**A**) Au/PATP/D−Phe and (**B**) Au/PATP/L−Phe enantiomeric discrimination systems. Compared *ρ*_CT_ profiles of (**C**) 1140, (**D**) 1388, and (**E**) 1433 cm^−1^ bands of PATP in the Au/PATP/D−Phe and Au/PATP/L−Phe systems.

In the case of the Au/PATP/D-Phe and Au/PATP/L-Phe systems, the same trend was observed for the intensity of the electrochemical potential-dependent SERS spectra of the two systems compared with the change trend of the Au/PATP system. With the potential scan started at −0.5 V, the intensity of the three b_2_ modes of PATP distinctly increased and then gradually decreased, with the maximum at around 0.1 V, as shown in Figure 5A,B. The change in the SERS spectral intensity in the enantiomeric discrimination systems indicated that the CT process was closely associated with the potential interference. The evident difference in the potential-dependent SERS spectra of the Au/PATP and the Au/PATP/Phe-enantiomer systems was the shift of the applied electrode potential corresponding to the maximum intensity of the b_2_ modes of PATP. In comparison with the two enantiomeric systems with inverse chirality, the discrepancies between the relative intensities of the b_2_ modes of the D- and L-Phe discrimination systems changed with the applied potential. The b_2_ modes of PATP at 1140, 1388, and 1433 cm^−1^ in the Au/PATP/D-Phe system were much stronger than those in the Au/PATP/L-Phe system at any potential over the potential range. Although the intensity difference between the two enantiomeric systems among the three b_2_ modes varies slightly, the trends of intensity difference variation were similar. We found the optimized potential condition for discrimination to be 0.1 V, at which the spectral discrepancies in the intensities of the b_2_ modes between the D- and L-Phe discrimination systems were remarkable. To further investigate such CT-induced enantioselective discrimination performance, the potential-dependent *ρ*_CT_ profiles of the three b_2_ bands of PATP in the Au/PATP/Phe-enantiomer systems were plotted. As shown in Figure 5C–E, the electric potential values corresponding to the maximum intensity of the b_2_ modes in the Au/PATP/Phe-enantiomer systems were shifted to 0.1 V, which were different from those in the Au/PATP system. The possible reason for the potential shift is that the modification of the Au/PATP substrate with enantiomers alters the molecular electronic structure of PATP and the CT state of the Au/PATP complex in the system [42]. In addition, the *ρ*_CT_ values of the b_2_ bands of PATP in the Au/PATP/D-Phe system were significantly higher than those in the Au/PATP/L-Phe system, indicating a more favorable CT process occurred in the D-Phe system than that in the L-Phe system [43]. The difference in the *ρ*_CT_ value between the two enantiomeric systems, which can determine the resolution for the chirality-label-free enantioselective discrimination strategy, largely depends on the applied electrode potentials and reaches its maximum at around 0.1 V. Therefore, regulation of CT in the enantiomeric systems by tuning the applied electrode potential can amplify the differential spectral signal to achieve optimum enantiomeric discrimination performance.

### 3.4. Electrochemical Potential-Regulated SERS Enantioselective Discrimination for Other Aromatic and Aliphatic Enantiomers

**Figure 6:**
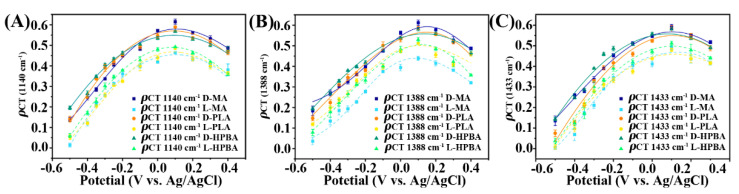
Compared *ρ*_CT_ profiles of (**A**) 1140, (**B**) 1388, and (**C**) 1433 cm^−1^ bands of PATP in the three aromatic enantiomeric discrimination systems.

To evaluate the general applicability of the potential-regulated enantioselective discrimination method, various chiral molecules, including aromatic and aliphatic molecules, were chosen to investigate the discrimination performance. Electrochemical SERS spectra of the enantioselective discrimination systems were measured for distinguishing three kinds of aromatic chiral molecules, MA, PLA, and HPBA molecules, whose molecular structures are analogous but only differ by a methyl group. The same trend of the potential-dependent *ρ*_CT_ profile for the b_2_ modes of the PATP in the three kinds of enantiomeric discrimination systems were observed, such as the Au/PATP/Phe discrimination system (Figure 6). While the remarkable difference among these three discrimination systems is the decrease in the discrepancy in the SERS spectral intensity between the corresponding two enantiomeric systems of each chiral molecule as the number of carbons in the chiral molecules increases. In other words, there is a higher discrimination resolution for MA enantiomers compared with PLA and HPBA molecules since the difference in the *ρ*_CT_ values of the two enantiomeric systems for MA is larger than that of the other two chiral molecules. Additionally, a tiny shift can be observed for the applied electrode potential corresponding to the maximum *ρ*_CT_ value of the enantiomeric discrimination system. With the increase in the number of carbons in the chiral molecules, the potential related to the maximum *ρ*_CT_ value decreased. It can be explained that increasing the number of carbons causes a decrease in the degree of conjugation and a change in the molecular electronic structure of the PATP/enantiomer complex, which affects the CT process in the Au/PATP/enantiomer system. Considering there is no large difference in the molecular weight among these three aromatic chiral molecules, the shift of the electrode potential related to the maximum *ρ*_CT_ value is extremely small.

**Figure 7:**
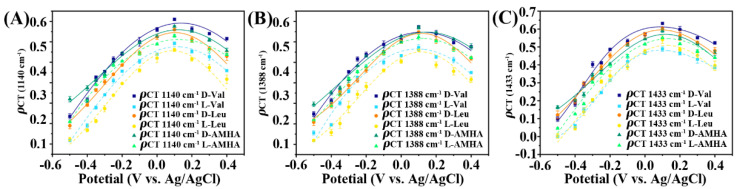
Compared *ρ*_CT_ profiles of (**A**) 1140, (**B**) 1388, and (**C**) 1433 cm^−1^ bands of PATP in the three aliphatic enantiomeric discrimination systems.

Except for the three aromatic chiral molecules, the potential-regulated SERS enantioselective discrimination method was also applied to the discrimination of the aliphatic chiral molecules containing Val, Leu, and AMHA, and similar changes in the spectral intensity and the *ρ*_CT_ profile were found in Figure 7. Notably, the shorter chain enantiomeric molecules, such as Val and Leu molecules, can be distinguished with a higher resolution by the potential-regulated enantioselective discrimination. In the case of AMHA discrimination, the discrepancy in the *ρ*_CT_ value between the Au/PATP/D-AMHA and the Au/PATP/L-AMHA is smaller than that obtained from the discrimination systems containing the other two aliphatic chiral molecules. It may be the reason for the change in the molecular electronic structure of the PATP/enantiomer complex with a nonrigid longer-chain aliphatic chiral molecule involved in the discrimination system. Nevertheless, effective enantioselective discrimination can be achieved by tuning the applied electrode potential for different chiral molecules discrimination. In light of the above electrochemical SERS results, the electrochemically potential-regulated SERS enantioselective discrimination exhibits powerful applicability for various chiral molecules with remarkable discrimination performance by regulating the applied potential to amplify the discrimination resolution.

## 4. Conclusions

A potential-regulated chirality-label-free enantioselective discrimination strategy for chiral small molecules was proposed based on CT-induced SERS spectroscopy, which not only retains the simplicity of the chirality-label-free methods but also possesses general applicability and high discrimination performance. The assembled Au NPs modified with the Raman probe PATP on the ITO-coated glass were used as the achiral discrimination substrates, which were further bonded with the target chiral molecules through the EDC/NHS coupling reaction to construct the Au/PATP/enantiomer discrimination system. The achiral Au/PATP substrates possess great SERS reproducibility, affording the possibility for reasonable enantiomeric discrimination. The Phe enantiomers were then distinguished by the differentiated SERS intensity of the b_2_ modes of PATP in the two enantiomeric systems, indicating the ability for CT-induced enantioselective discrimination. The electrochemically potential-dependent SERS spectra for distinguishing Phe enantiomers also provided evidence that CT resonance was involved in the enantioselective discrimination. The *ρ*_CT_ profiles of the discrimination systems manifested that the discrimination resolution was closely associated with the applied electrode potential, and the chiral signal amplified enantiomeric discrimination was achieved by regulating the applied potential at around 0.1 V. In addition, various chiral molecules, including aromatic and aliphatic molecules, were chosen to evaluate the universality of this CT-induced chirality-label-free enantioselective discrimination method, and to further investigate the relationship between the potential-dependent discrimination performance and the molecular structure. As for the three aromatic chiral molecules, MA, PLA, and HPBA, good discrimination performance was obtained by tuning the applied potential in the enantiomeric systems. In the case of the aliphatic chiral molecules, only the shorter chain enantiomeric molecules, Val and Leu molecules, can be distinguished with higher resolution by the potential-regulated SERS spectroscopy. One of the suggested reasons is that the aliphatic molecules, especially the longer chain molecule AMHA, do not cause the delocalization of electrons to promote the CT process like the aromatic molecules. Another reason is that the longer chain aliphatic molecule may influence the molecular electronic structure of the PATP, which affects the CT process in the discrimination system. Our work demonstrated the relationship between discrimination performance and the electrochemically potential-regulated CT process and thus developed the potential-regulated chirality-label-free enantioselective discrimination strategy that possessed general applicability for various chiral molecules with high discrimination resolution. The potential-regulated chirality-label-free enantioselective discrimination strategy we proposed has potential applications not only in the analysis of amino acids generated from asymmetric reactions with high discrimination resolution but also in the estimation of the levels of the amino acids in human bodies that are associated with diseases.

## Data Availability

No new data were created or analyzed in this study. Data sharing is not applicable to this article.

## Supplementary Materials

The following supporting information can be downloaded at: https://www.mdpi.com/article/10.3390/bios13040471/s1, Figure S1: (A) SERS spectrum of the Au/PATP substrate. SERS mapping images measured from the Au/PATP substrate with a randomly selected area of 10 × 10 µm^2^. mapping images were integrated by the SERS bands of PATP at (B) 1705 and (C) 1140 cm^−1^. Figure S2: CV curves of the Au substrate, Au/PATP, and Au/PATP linked with D- and L-Phe enantiomers. Figure S3: Potential-dependent SERS spectra of the Au/PATP substrate.
