# Supplementary material for: A Charge-Transfer-Induced Strategy for Enantioselective Discrimination by Potential-Regulated Surface-Enhanced Raman Scattering Spectroscopy
Source: Biosensors (Basel). 2023 Apr 12;13(4):471. doi: 10.3390/bios13040471 (PMC10136649; doi:10.3390/bios13040471)
Supplement: Supplementary file 1 [file biosensors-13-00471-s001.zip › biosensors-2275157-supplementary.pdf]

biosensors

MDPI

*Article*

# A Charge-Transfer-Induced Strategy for Enantioselective Discrimination by Potential-Regulated Surface-Enhanced Raman Scattering Spectroscopy

**Yue Wang [1], Yucong Liu [1], Chunyu Ren [1], Ruofei Ma [1], Zhangrun Xu [1,*] and Bing Zhao [2,*]**

[1] Department of Chemistry, College of Sciences, Northeastern University, Shenyang 110819, China
[2] State Key Laboratory of Supramolecular Structure and Materials, Jilin University, Changchun 130012, China
[*] Correspondence: xuzr@mail.neu.edu.cn (Z.X.); zhaob@jlu.edu.cn (B.Z.);
Tel./Fax: +86-24-83867659 (Z.X.); +86-431-85168473 (B.Z.)

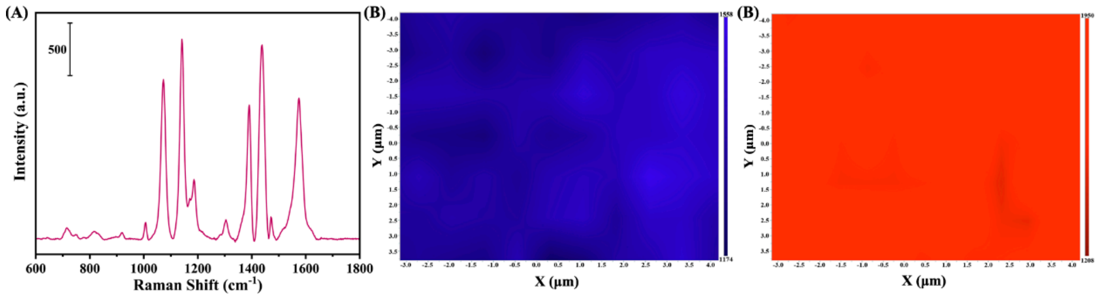


**Figure S1.** (**A**) SERS spectrum of the Au/PATP substrate. SERS mapping images measured from the Au/PATP substrate with a randomly selected area of 10 × 10 μm$^2$. mapping images were integrated by the SERS bands of PATP at (**B**) 1705 and (**C**) 1140 cm$^{-1}$.

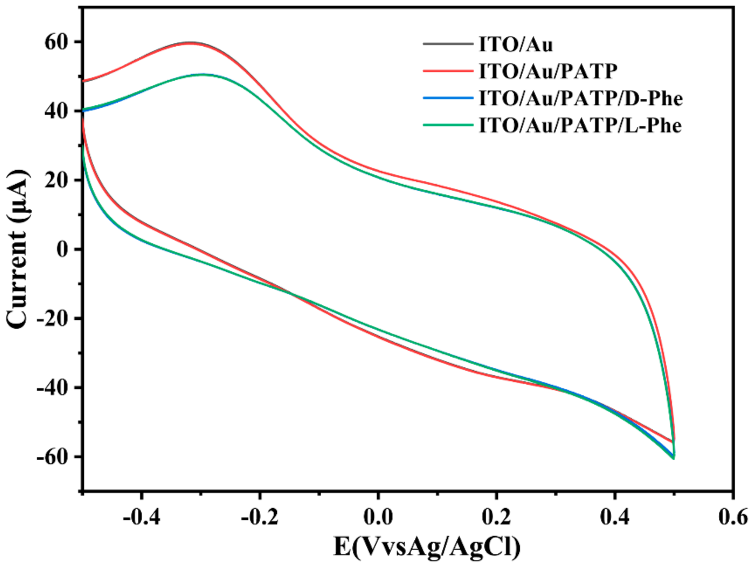


**Figure S2.** CV curves of the Au substrate, Au/PATP, and Au/PATP linked with D- and L-Phe enantiomers.

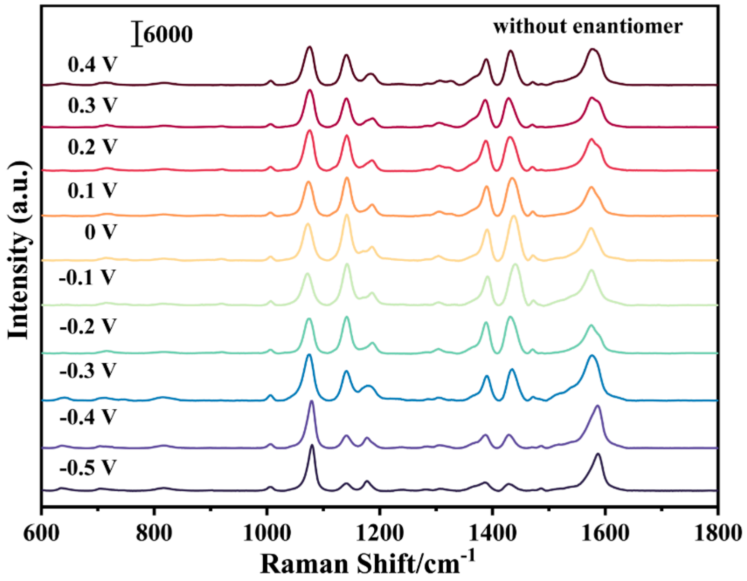


**Figure S3.** Potential-dependent SERS spectra of the Au/PATP substrate.
